# MTSS1: beyond the integration of actin and membrane dynamics

**DOI:** 10.1007/s00018-024-05511-w

**Published:** 2024-12-03

**Authors:** Liudmila Matskova, Shixing Zheng, Elena Kashuba, Ingemar Ernberg, Pontus Aspenström

**Affiliations:** 1 Department of Microbiology, Tumor and Cell Biology, Karolinska Institutet, Stockholm, FE 280, 17177 Sweden; 2 RE Kavetsky Institute of Experimental Pathology, Oncology and Radiobiology of National Academy of Sciences of Ukraine, Kyiv, 03022 Ukraine; 3 ENT Institute, Department of Otorhinolaryngology, Eye & ENT Hospital, Fudan University, Shanghai, 200031 China; 4 Rudbeck Laboratory, Department of Immunology, Genetics and Pathology, Uppsala University, Uppsala, 75185 Sweden

**Keywords:** I-BAR, Invasion, Metastasis, Tumor suppressor, Membrane architecture, Lipid metabolism

## Abstract

MTSS1 is a ubiquitously expressed intracellular protein known mainly for its involvement in basic cellular processes, such as the regulation of actin organization and membrane architecture. MTSS1 has attracted much attention for its role as a tumor suppressor, being absent or expressed at reduced levels in advanced and metastasizing cancers. Occasionally, MTSS1 is, instead, upregulated in metastasis and, in some cases, even in primary tumors. In addition to these well-established functions of MTSS1 linked to its I-BAR- and WH2-domains, the protein is involved in modulating cell–cell contacts, cell differentiation, lipid metabolism, and vesicle formation and acts as a scaffolding protein for several E3 ubiquitin ligases. MTSS1 is classified as a housekeeping protein and is never mutated despite the several pathologic phenotypes linked to its dysregulation. Despite MTSS1’s involvement in fundamental signaling pathways, MTSS1 gene ablation is not ubiquitously lethal, although it affects embryonic development. Due to MTSS1´s involvement in many seemingly disparate processes, with many cases lacking mechanistic explanations, we found it timely to review the recent data on MTSS1’s role at the cellular level, as well as in health and disease, to direct further studies on this interesting multifunctional protein.

## Introduction

MTSS1 (metastasis suppressor 1), also known as missing in metastasis (MIM), is expressed at varied levels in most human tissues and has multiple functions; playing important roles in carcinogenesis and cell proliferation and differentiation. It was first identified as a metastasis suppressor, being absent or expressed at low levels in cell lines originating from metastatic bladder carcinoma [1]. Subsequently, MTSS1 was shown to be downregulated in many other types of cancers [2].

MTSS1 expression is relatively low in breast cancer [3], glioblastoma [4], diffuse large B-cell lymphoma (DLBCL) [5], and nasopharyngeal carcinoma [6] compared with control tissue from healthy subjects. Higher levels of MTSS1 expression were associated with better prognosis in patients with epithelial cancers [2]. *MTSS1* has been suggested to serve as a sonic hedgehog (SHH)-responsive gene being involved in the regulation of glioma-associated oncogene homolog 1 (GLI1)-dependent transcription upon carcinogenesis [7]. MTSS1 contains sequence motifs implying its action as a scaffolding protein, binding to multiple partners to regulate complex biological processes. This protein’s main characteristic is linked to its ability to directly interact with many cellular factors active in diverse pathways.

**Fig. 1:**
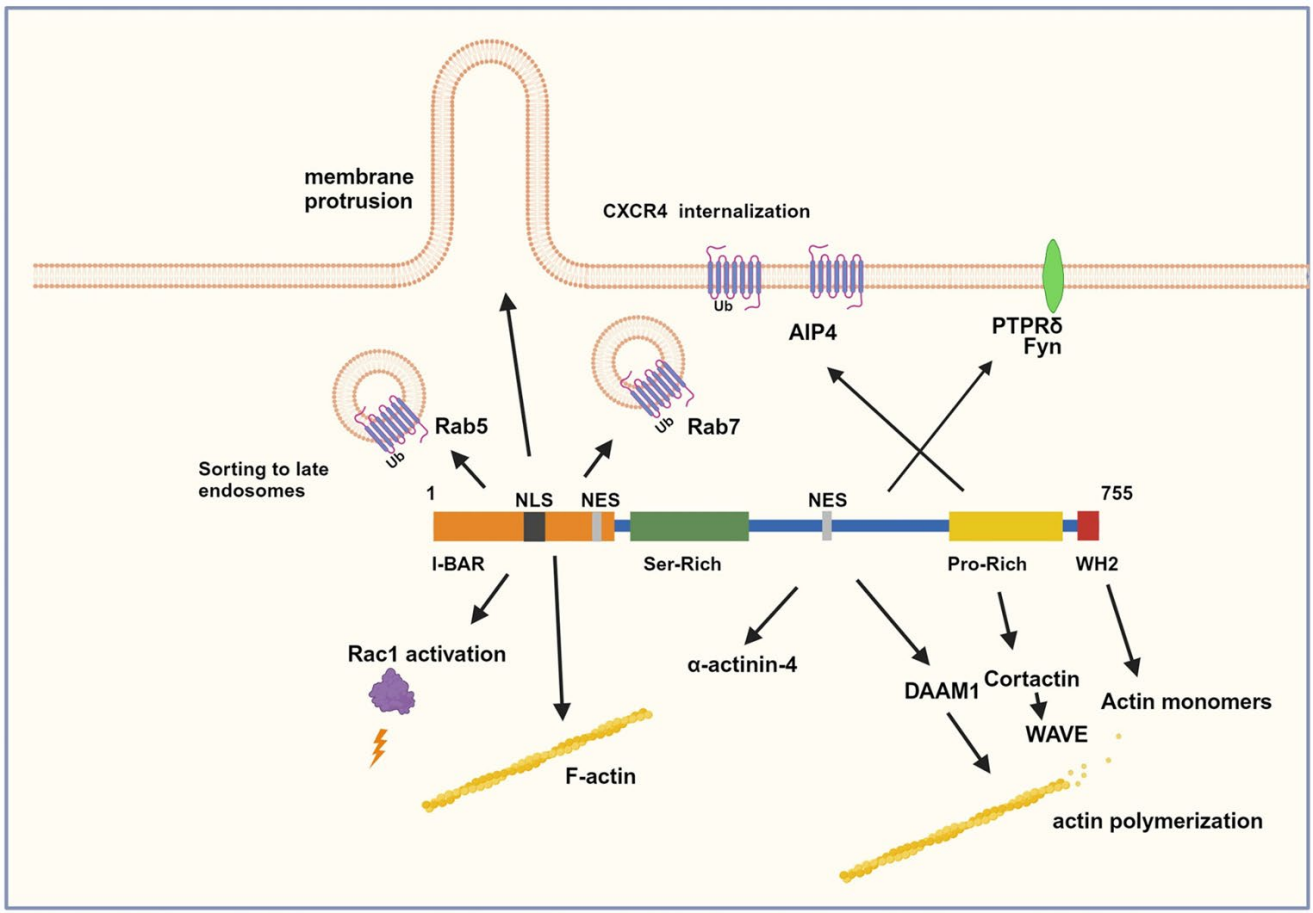
A schematic representation of MTSS1’s structure and functions. The I-BAR domain of the MTSS1 protein is involved in actin bundling and lamellipodia formation. The WH2 domain binds to monomeric actin

MTSS1 has four well-documented biochemical functions: (1) it induces membrane protrusions (lamellipodia and filopodia) by a direct interaction between the I-BAR domain and membrane lipids; (2) it modulates actin dynamics by slowing down actin nucleation and filament elongation via the WH2 domain; (3) it modulates the accumulation of F-actin at cell–cell borders or at filopodia via the I-BAR domain; and (4) it acts as a scaffolding protein, bringing together many accessory proteins, particularly in the context of cytoskeletal modulation and protein degradation (Fig. 1). Although these four functions are central to cell dynamics, studies on the functions of MTSS1 have so far been far from conclusive.

In addition, it was recently reported that MTSS1 levels were elevated upon adipogenic differentiation of mesenchymal cell lines and primary bone marrow stromal cells in vitro [8]. This was due to the binding of MTSS1 to FYN, a member of the Src family of tyrosine kinases (SFKs), and to protein tyrosine phosphatase receptor-δ (PTPRD). Moreover, MTSS1 was found to be upregulated during osteoblast differentiation, thereby promoting osteogenic differentiation from marrow stromal progenitor cells [9]. As a result, the Src pathway was inhibited, while MTSS1 induced the canonical Wnt signaling in these cells.

MTSS1 is emerging as a central player in cell biology, modulating cell migration, cell membrane dynamics, differentiation, cell–cell connectivity, and intracellular protein trafficking (Fig. 1). The functions of MTSS1 in a variety of cellular processes must be better understood to understand this protein’s sentinel surveillance role in counteracting metastasis and possibly additional cancer hallmarks. MTSS1 seems to have roles in carcinogenesis, the control of cell proliferation and differentiation, B-cell-mediated immunity, and cellular signaling and metabolism [10]. In this article, we aim to review the currently known functions of this multifaceted protein.

## The I-BAR family of proteins

I-BAR domain, also known as IRSp53/MIM homology domains (IMD), represents a subgroup of the larger group of Bin/amphiphysin/Rvs (BAR) domains, which bind and influence the curvature of lipid bilayers [11, 12]. The I-BAR proteins represent a subgroup of a much larger group of proteins called BAR proteins, which, in addition to I-BAR proteins, also include N-BAR, F-BAR, PX-BAR and BAR-SH3 proteins, to mention the most common members of this superfamily [13], The I-BAR family of proteins consists of five members: MTSS1; insulin receptor tyrosine kinase substrate of 53 kDa (IRSp53) (also known as brain-specific angiogenesis inhibitor 1-associated protein 2 (BAIAP2)); insulin receptor tyrosine kinase substrate (IRTKS) (also known as BAIP2-like protein 1 (BAIAP2L1)); actin-bundling protein with BAIAP2 homology (also known as ABBA or MTSS2); and BAIAP2-like protein 2 (BAIAP2L2) (also known as FLJ22582 or Pinkbar) [14]. MTSS1 and MTSS2 form one subgroup, while IRSp53, IRTKS, and BAIAP2L2 form another. The most decisive domains of MTSS1, as well as for MTSS2, are the N-terminal I-BAR domain and the C-terminal WASP homology 2 (WH2) domain. In contrast, proteins belonging to the IRSp53 subgroup have, in addition to the N-terminal I-BAR domains, an SH3 domain in the other half of the proteins. A WH2-like motif has been described in the C-termini of IRSp53 proteins. However, structural modelling does not support the notion that this motif can attain a WH2-like fold. MTSS1 is widely expressed in human tissue (https://www.proteinatlas.org/ENSG00000170873-MTSS1*)*, which is the case also for MTSS2, but, compared to MTSS1, MTSS2 has significantly higher expression in the brain (https://www.proteinatlas.org/ENSG00000132613-MTSS2*).*

**Fig. 2:**
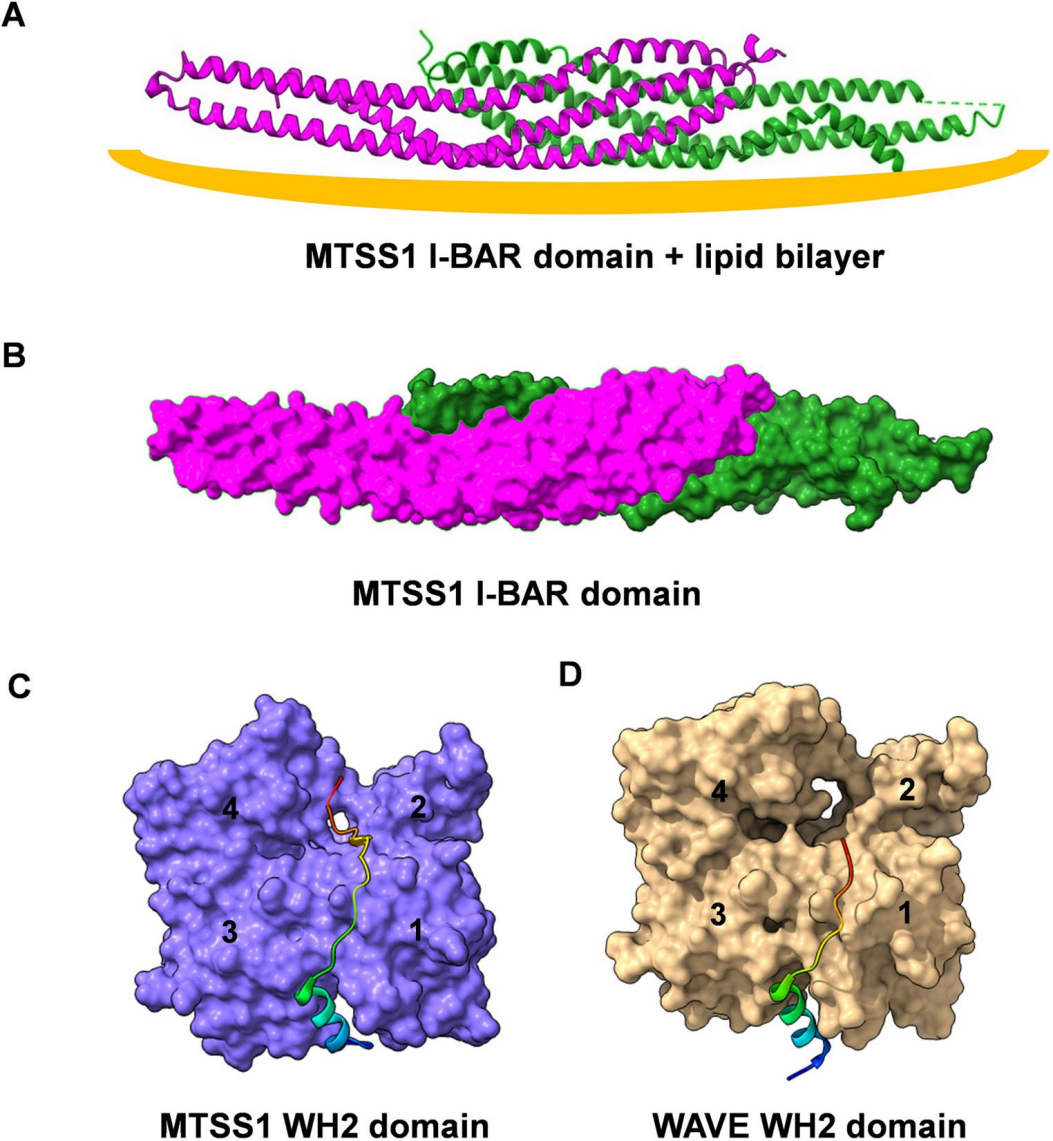
(**A**) Structure of MTTS1 I-BAR domain dimer encompassing amino acid residues 1-234 together with a lipid bilayer (marked in orange). (**B**) Structure of the MTSS1 I-BAR domain alone. (**C**) Structure of MTSS1 WH2 domain in complex with α-actin showing interaction with all four actin subdomains. (**D**) Structure of WAVE WH2 domain in complex with α-actin showing interaction with actin subdomains 1 and 3. Images were generated with USCF ChimeraX software (http://www.rbvi.ucsf.edu/chimera. http://www.rbvi.ucsf.edu/chimerax/version: 1.2.5 (24 May 2021))

I-BAR domains are formed by three extended α-helices, which dimerize and form 180 Å extended Zeppelin-shaped structures (Fig. 2A, B). Positively charged amino acid residues are concentrated at the ends of the I-BAR domains and confer binding to lipid bilayers [15, 16]. Unlike the classical BAR domains, which trigger invaginations of lipid membranes, the I-BAR domains induce the formation of protrusions, hence the name inverse BAR (I-BAR).

## MTSS1 is involved in actin remodeling

MTSS1 has important roles in regulating actin dynamics via several domains with actin-binding capacity. The WH2 domain at its C-terminus has been shown to bind actin monomers with high affinity [17, 18]. The WH2 domain of MTSS1 was found to be more extended compared with the WH2 motifs present in the WASP family of proteins. The 3D structure in the complex of G-actin and the actin-sequestering protein DNAse I shows that the WH2 domain of MTSS1 interacts with all four subdomains of actin as opposed to the WH2 domain of the WASP family of proteins, which binds to a cleft between actin subdomains 1 and 3 (Fig. 2C, D) [15]. In vitro experiments with an isolated GST fusion protein encompassing this WH2 domain showed that it slowed down actin nucleation and filament elongation [17, 18]. In contrast, the I-BAR domain of MTSS1 was suggested to possess actin-bundling activity in vitro using an isolated recombinant protein although this interpretation has been challenged in later studies claiming the bundling activity to be an artifact [11, 19]. However, ectopic expression of the MTSS1 I-BAR domain in many cell types result in the formation of side-by-side accumulation of actin filaments [6, 20, 21]. The underlying mechanisms are not clear; however, mutations in the MTSS1 I-BAR domain that disrupt its membrane-binding capacity abolish its actin-bundling ability [22].

Interestingly, the I-BAR domain was found to bind to Rac1, a member of the Rho family of small GTPases, and promote the formation of activated GTP-bound Rac1 [22]. The ability to bind and activate Rac1 is shared with the MTSS2 but, in contrast to MTSS1, MTSS2 does not possess a capacity to trigger the formation of higher order structure of actin filaments [23]. The I-BAR domain shows no resemblance to any domains with RhoGEF activity, so it is unclear how the Rac1 activation is catalyzed. IRSp53 binds to the Rac1-specific RhoGEF protein Tiam1, so the Rac1 activation may be mediated by Tiam1 or another interacting RhoGEF [24].

Ectopic expression of full-length MTSS1 results in lamellipodia formation or the accumulation of F-actin at cell–cell borders, depending on the cell type [17, 18, 22]. The current view is that MTSS1 acts as a scaffold protein, bringing together many accessory proteins to modulate cytoskeletal regulation. These interaction partners often bind to the central portion of MTSS1. The Arp2/3-regulating protein cortactin binds via its SH3 domain to the proline-rich motifs of MTSS1, and MTSS1 can thereby potentiate cortactin-mediated actin polymerization [25]. The central portion of MTSS1 binds and recruits the actin-bundling protein α-actinin 4 to cell areas undergoing membrane ruffling [26]. The binding site for PTPRD in MTSS1 is also localized in the central parts of MTSS1. The PTPRD interaction facilitates MTSS1-dependent actin reorganization and is also required for PTPRD localization to the plasma membrane [20]. Moreover, MTSS1 was found to bind the WASP family protein WAVE-2 and participate in the formation of lamellipodia-like protrusions at epithelial cell–cell junctions [27].

Some of the central functions of MTSS1 have been established via studies in neuronal cells. MTSS1 was found to have a specific role in the formation of so-called dendritic filopodia, i.e., precursors to dendritic spines, which function as contact sites for neighboring axons [28]. MTSS1 was shown to collaborate with the ENA/Vasp family protein EVL in the initiation and elongation of dendritic filopodia in a process involving the formation of branched actin filaments via the activation of the Arp2/3 complex [29]. The Arp2/3 activation is, in turn, mediated via the interaction between MTSS1 and N-WASP [30]. MTSS1 can also negatively regulate dendritic spine elongation through the interaction with diaphanous-related formin DAAM1 [31].

In summary, these observations imply that MTSS1 can function as a modulator of dendritic filopodia formation and maturation. Another function related to filopodia function is the formation of a group of extracellular vesicles called filopodium-derived vesicles. MTSS1 has a role in the formation and shedding of these particles from the tips of filopodia. These vesicles have been shown to contain signaling molecules, for instance, Rac1, which can activate recipient cells [32].

## Role of MTSS1 during embryonic development

There are several studies on MTSS1 knockout in mice. Mice lacking MTSS1 generally survive the embryonic stage, although they are not healthy. Saarikangas et al. found that the full-length MTSS1 protein was dispensable for embryonic development [33]. However, surviving embryos had bone abnormalities in cardiac development and end-stage renal failure [33]. Another study focusing on neurological effects revealed enlarged brain ventricles and a decreased cortical volume, which manifested as learning defects, alterations in anxiety levels, and reduced dominant behavior, suggesting a deficiency in motor coordination and pre-pulse inhibition [34]. Mice lacking MTSS1 also have enlarged spleens, and lacking MTSS1 triggers lymphomagenesis [8]. In contrast, a study in another vertebrate model, Medaka (also known as Japanese rice fish), showed that a significant proportion of CRISPR-mediated MTSS1 knockout embryos were dead four days after fertilization [35]. It is unclear why MTSS1^-/-^ mice survive to a much higher degree. The reason could be linked to differences between animal models, experimental approaches, or compensating expressions of other I-BAR proteins in MTSS1^-/-^ embryos.

## Regulation of MTSS1 expression

The regulation of MTSS1 expression remains an open question, despite the frequently observed changes in MTSS1 expression levels in different tissues and different cancers and the role of MTSS1 expression levels in carcinogenesis. To this end, the epigenetic control of MTSS1 expression has been most studied. For instance, the MTSS1 promotor region is heavily methylated in glioblastoma [36]. Decreased levels of *MTSS1* mRNA due to promotor methylation were also reported for bladder urothelial carcinoma [37], prostate cancer [38], and chronic myeloid leukemia [39]. Moreover, *MTSS1* down-regulation was associated with elevated expression of miR-96 in prostate cancer [40].

An extensive bioinformatics analysis was performed to identify transcription factors (TFs) and their putative binding sites in promotor and enhancer regions of the *MTSS1 gene.* GATA1,2 and Myc are the most likely TFs involved in the control of MTSS1 gene expression [41]. However, the predicted TF regulation of *MTSS1* transactivation needs to be validated in in vivo experiments.

Epigenetic control serves as another way to regulate MTSS1 expression. To this end, MTSS1 downregulation by hypermethylation is a mechanism associated with the progression of gastric [42], glioblastoma [36], prostate cancers [43] and lung giant-cell carcinoma [44]. Several microRNAs (miR-96 [40, 45], miR-182 [46], miR-29a, miR-411 [47], miR-23a [5, 48], miR-15b [49], and miR-135b [50]) downregulate MTSS1,thereby promoting tumor growth, invasion, and metastasis in prostate, hepatocellular, non-small cell lung, osteosarcoma, and colorectal cancers.

## MTSS1 acts as a scaffold protein for E3 ubiquitin ligases

MTSS1 protein levels in cancer cells can be reduced via proteasomal degradation. The SKP1, cullin-1 (CUL1), and F-box protein (SCF) E3 ubiquitin ligase complex can target MTSS1 for ubiquitination and subsequent degradation by the 26 S proteasome. The β-transducin repeat-containing protein (β-TRCP, also called BTRC) constitutes the F-box protein in this SCF complex. The depletion of either CUL1 or β-TRCP resulted in an increased MTSS1 level [51]. The researchers identified an evolutionally conserved phosphodegron motif (DSGXXS) in MTSS1, i.e., a motif that promotes interaction with ubiquitin ligases upon phosphorylation. The serine residue 322 in this motif, which is responsible for the interaction between the phosphorylated MTSS1 and β-TRCP, can be phosphorylated by casein kinase Iδ (CKIδ), thereby triggering MTSS1 ubiquitination and its subsequent degradation. Importantly, introducing wild-type MTSS1 or a mutated non-degradable MTSS1 (MTSS1/S322A) into breast or prostate cancer cells with low wild-type MTSS1 expression significantly inhibited cellular proliferation and migration. Moreover, ectopically expressed MTSS1/S322A inhibited cell proliferation and migration more efficiently compared with wild-type MTSS1, indicating a fast turnover of the MTSS1 protein in vivo and/or an altered post-translational regulation in tumor cells [51].

Another control of MTSS1 was proposed by the observed interaction between the LINC00491 long non-coding RNA and MTSS1 in lung adenocarcinoma (LUAD) [52]. LINC04091 was significantly upregulated in LUAD and positively correlated with poor survival, enhanced proliferation, migration, and invasion of cancerous cells. Increased expression of LINC00491 was found to correlate with reduced MTSS1 protein levels without affecting the mRNA level. LINC00491 triggered the ubiquitination and degradation of MTSS1. In addition, LINC00491 expression resulted in the activation of the Wnt/β-catenin-signaling pathway, increasing GSK3β phosphorylation.

Enforced expression of MTSS1 reversed the upregulation of the Wnt-signaling pathway activated by LINC00491 [52]. The exact mechanism underlying the LINC00491-induced MTSS1 ubiquitination requires clarification. Following these observations, an inhibitory effect of MTSS1 on Wnt/β-catenin signaling was observed in ovarian cancer cells, where it mediated GSK3β phosphorylation at serine residue 9, thereby inhibiting its activity [53]. In contrast, increased MTSS1 expression instead stimulated Wnt/β-catenin signaling during mouse osteoblast differentiation and bone development [9].

It was suggested that the antagonism between MTSS1 and the Src family tyrosine kinases could explain the observed contradictory effects of MTSS1 on Wnt/β-catenin signaling [54, 55]. Src exhibits either stimulatory or inhibitory effects on the canonical Wnt signaling, depending on the cellular context [56]. Src promotes tumor progression (at least, partially) in tumor cells by enhancing Wnt/β-catenin signaling [57, 58]. In contrast, Src exhibits an inhibitory effect on the Wnt/β-catenin signaling pathway in non-tumor cells, such as mouse osteoblasts and embryonic fibroblasts [9, 59]. Wnt/β-catenin signaling is blocked when Src phosphorylates the low-density lipoprotein receptor-related protein 6 (LRP6) at conserved tyrosine residues, which results in LRP6 removal from the cell surface and the disruption of LRP6 signalosome formation [59]. Most likely MTSS1 competes for the interaction with the kinases responsible for phosphorylating the inhibitory and/or activating sites on GSK3β and Src.

MTSS1 is also involved in the regulation of the immune checkpoint protein programmed death-ligand 1 (PD-L1) in LUAD. MTSS1 forms a complex with PD-L1 and AIP4, which promotes the mono-ubiquitination of PD-L1 on lysine residue 263 [60]. This ubiquitination of PD-L1 targets the protein for lysosomal degradation. Mutating lysine 263 into an arginine residue abolishes PD-L1 ubiquitination. Oncogenic activation of the EGF/RAS pathway, e.g., by activating EGFR^T790M^ or KRAS^G12C^ mutations, results in decreased MTSS1 expression followed by PD-L1 stabilization. Thus, strategies aiming to increase MTSS1 expression or stabilize the protein could be favorable for immune checkpoint therapy to enhance anti-tumor immunity.

Studies on ring finger protein 180 (RFN180) presented another link between E3 ligases and MTSS1. Promoter methylation of *RNF180* was detected in 76% of primary gastric cancers and 55% of intestinal metaplasia but in none of 23 normal gastric tissues. Methylated *RNF180* DNA was detected in the plasma of 56% of gastric cancer patients but not in healthy controls [61]. Re-expression of RNF180 resulted in apoptosis and the suppression of cell proliferation, presumably mediated via the upregulation of three genes: *MTSS1*,* CDKN2A*, and the proapoptotic protein *TIMP3* [61].

## MTSS1 in inflammation

MTSS1 has been implicated in the regulation of inflammation and, more specifically, the degradation of the p65 component of NF-κB. The mechanism appears to involve the interaction between MTSS1 and the E3 ligase RanBP2-type and C3HC4-type zinc finger-containing 1 (RBCK1). This mediates RBCK1-mediated p65 ubiquitination and degradation, which suppresses the NF-κB signaling pathway and tumorigenesis. In addition, the β-actin-related protein actin beta-like 2 (ACTBL2) competes with RBCK1 for MTSS1 binding, but it has an opposing effect on tumorigenesis compared with MTSS1. MTSS1 downregulates p65, but ACTBL2 expression results in p65 stabilization.

Thus, MTSS1 acts as a scaffold protein for the ubiquitin–proteasome system, which regulates key signaling pathways such as NF-κB [62]. This is a mechanism of the MTSS1-mediated suppression of tumor-initiating cells (TICs, also called cancer stem-like cells) seen in breast cancer mouse models. *MTSS1* ablation enhanced the mammary epithelial TIC subpopulation in both luminal and basal-like breast cancer mouse models [62]. Simultaneously, it was reported that high MTSS1 expression promoted inflammatory brain injuries after intracerebral hemorrhage (ICH) by enhancing inflammatory cytokine secretion and targeting miR-709 expression [63]. MTSS1 was suggested to be an inflammation driver, together with thioredoxin-interacting protein and ubiquitin-specific protease 2 [64].

## MTSS1’s role in the regulation of receptor internalization

BAR-containing proteins serve important roles in regulating endocytosis, exocytosis, and membrane dynamics [65]. As such, MTSS1 also plays a role in endocytosis, as documented in the internalization of the C-X-C motif chemokine receptor 4 (CXCR4). This chemokine receptor and its ligand CXCL12 (also known as stromal-derived-factor 1 (SDF1)) are involved in the regulation of the chemotactic response in lymphocytes [66]. Using HeLa cells as a model system, it was found that MTSS1 forms a complex with CXCR4, together with the E3 ubiquitin ligase AIP4 (also known as ITCH) in response to activation with CXCL12. MTSS1 overexpression promoted CXCR4 ubiquitination, inhibited the cellular response to CXCL12, caused the accumulation and aggregation of multivesicular bodies (MVBs) in the cytoplasm, and promoted CXCR4 sorting into MVBs in a manner dependent on binding to AIP4. The sorting of the internalized CXCR4-containing vesicles required the involvement of Rab7, which interacts with the I-BAR domain of MTSS1 (Fig. 1) [67]. MTSS1 also interacts with Rab5, but the timing of the interactions with the two Rabs differ. In response to CXCL12 stimulation, the CXCR4-containing endosomes first interact with Rab5 and then with Rab7 before sorting into MVBs [68]. The involvement of MTSS1 in the formation and traffic of intracellular vesicles has already found practical applications in experimental drug delivery in cancer treatment using magnetic nanoparticles [69]. The internalization of magnetic nanoparticles increased in an MTSS1-dependent manner, providing a basis for their functional application in cell physiology [70].

Studies on the Drosophila orthologue of MTSS1, called DMIM, have revealed an inhibitory function for the protein in endocytosis by a mechanism involving sequestering of cortactin, which normally forms a functional complex with the CD2-associated protein (CD2AP). The CD2AP/cortactin complex receives signaling cues that provides directional guidance for cell migration [46]. DMIM counteracts CD2AP/cortactin signaling and prevents the cell from sensing guidance cues. Thus, absence of the negative influence on the CDAP2/cortactin complex could be one mechanism behind the increased migratory capacity of tumor cells in late-stage cancers, where MTSS1 is silenced [46].

MTSS1 is involved in adipogenic differentiation, and several MTSS1-interacting partners are involved in lipid metabolism, for example, PTPRD [8]. PTPRD is a receptor for the adipokine asprosin, a glucose-raising protein hormone [71]. MTSS1 might be involved in the internalization of PTPRD, similar to its role in the internalization of CXCR4 and PD-L1, thus protecting cells from increased glucose uptake and blocking lipid deposits. MTSS1’s contribution to the movement and interactions of intracellular structures, such as vesicles, lipid droplets, and mitochondria, is an interesting future field of study [63].

## MTSS1 in cancer

The original findings of MTSS1 being absent or expressed at a low level in metastatic bladder carcinoma cell lines resulted in the dominant view that a lack of MTSS1 was a predictive factor for metastatic cancers [1]. This first observation was followed by many others demonstrating reduced MTSS1 levels in clinical samples. However, MTSS1’s role in tumors is more complex than originally thought. Some studies have shown that MTSS1 is also reduced in non-metastatic tumor cell lines and samples while being overexpressed in tumors compared with normal tissue. This apparent dichotomy has led to questions regarding the exact role of MTSS1 in tumor progression.

The dysregulation of MTSS1 and prognostic outcomes of cancer patients are summarized in Table 1. The general trend observed in these studies is that high MTSS1 expression is associated with low-grade tumors in epithelial tumors, while low MTSS1 expression is correlated with advanced TNM stages and metastasis. Only a small number of cases show a correlation between MTSS1 overexpression and metastasis [72–77]. The association of low MTSS1 expression with advanced cancer stages and metastasis highlights its significance as a prognostic marker for cancer progression. Thus, MTSS1 downregulation in cancer related to metastatic risk is more commonly observed than the few cases of MTSS1 upregulation related to metastasis (Table 1).

**Table 1:** Summary of MTSS1 expression and its prognostic outcomes in cancer patients

Cancer Type	(RNA/Protein) Expression in Cancer	Prognostic Marker
Glioblastoma	Decreased [4, 36]	Favorable [4, 36]
Nasopharyngeal carcinoma	Decreased [6]	Favorable [6]
Esophageal squamous cell carcinoma	Decreased [78]	Favorable [78]
Gastric cancer	Decreased [42, 79, 80]	Favorable [79]
Hepatocellular carcinoma (hepatitis B-related)	Increased [72]	Unfavorable [72]
Hepatocellular carcinoma	Decreased [46] Increased [73, 74]	Unfavorable [74]
Cholangiocarcinoma	Decreased [81, 82]	Favorable [81, 82]
Pancreatic cancer	Decreased [83, 84]	Favorable [83, 84]
Colorectal cancer	Decreased [49, 50, 85] Increased [75]	Favorable [49] Unfavorable [75]
Lung adenocarcinoma	Decreased [86]	Favorable [86]
Non-small cell lung cancer	Decreased [87] Increased [76]	Favorable [76, 88]
Kidney cancer	Decreased [89]	
Bladder cancer	Decreased [90, 91]	Favorable [91, 92]
Prostate cancer	Decreased [40, 43, 93]	
Breast cancer	Decreased [3, 45, 94, 95]	Favorable [3, 94–97]
Breast cancer (triple-negative)	Decreased [98]	Favorable [98]
Cervical cancer	Increased [77]	
Melanoma		Unfavorable [99]
Acute myeloid leukemia	Decreased [100, 101]	Favorable [100, 101]
Osteosarcoma	Decreased [47]	

Understanding the role of MTSS1 in cancer biology and the mechanism is crucial for employing it in therapeutic strategies. MTSS1’s antimetastatic mechanisms remain unclear. MTSS1 protein levels in cancer cells can be reduced via proteasomal degradation. MTSS1 also interacts with proteins like caveolin-1 [74], Rab GTPases [67], and RBCK1 [62], influencing the EGFR, CXCR4, and NF-κB signaling pathways, and is positively regulated by PTEN, inactivating the PI3K/AKT pathway [80, 85]. In addition, MTSS1 has been suggested to be involved SHH signaling [3, 79]. However, this concept has been disputed in a study by Saarikangas et al., which could not find evidence that MTSS1 could have a role in SHH-dependent transcription [33]. A firmly established tumor suppressor function of MTSS1 is to control actin dynamics and cell adhesion by affecting cancer cell motility [48, 67, 97, 99]. MTSS1 was shown to suppress nasopharyngeal carcinoma (NPC) cell migration and invasion in vitro through cytoskeletal remodeling at cell–cell borders and the assembly of E-cadherin/β-catenin in adherens complexes [6]. Furthermore, it was found that the I-BAR domain of MTSS1 is both necessary and sufficient to promote the formation of E-cadherin/β-catenin/F-actin-mediated cell adherens junctions [6].

Knockout mouse experiments have provided further arguments for MTSS1’s role in cancer beyond the control of actin dynamics. Approximately 80% of *MTSS1*^-/-^ mice developed tumors that resembled DLBCL in 1–2 years [102]. The *MTSS1*^-/-^ mice showed an abnormal distribution of B cells in lymphoid organs: a lower proportion in the spleen and a larger proportion in the bone marrow and peripheral blood. The IgM response was partially inhibited upon immunization with T-cell-independent antigens in these mice compared with wild-type mice. In parallel, *MTSS1*^-/-^ mice demonstrated undisturbed B-cell development and a normal B-cell compartment composition in the periphery [10]. MTSS1 levels in acute B-lymphocytic leukemia and lymphomas were low or below the threshold of detection in primary B-cells. The antigen response of the B-cell-receptor (BCR) pathway in B-cells of *MTSS1*^-/-^ mice was disturbed. Nevertheless, these B cells were metabolically more active in response to lipopolysaccharide (LPS) or CpG oligodeoxynucleotides (CpG, short synthetic single-stranded DNA molecules containing CpG motifs).

The mechanisms underlying the anticancer functions of MTSS1 are not yet fully understood; however, several mechanisms have been identified in various types of cancer. For instance, MTSS1 suppresses the migration and invasion of nasopharyngeal carcinoma cells in vitro by facilitating cytoskeletal remodeling at cell–cell junctions and promoting the assembly of E-cadherin, β-catenin, and F-actin in adherens junctions. Notably, the I-BAR domain of MTSS1 is both necessary and sufficient for restoring the formation of E-cadherin/β-catenin/F-actin-mediated adherens junctions [6]. In breast cancer, MTSS1 interacts with the E3 ligase RBCK1, enhancing RBCK1-mediated ubiquitination and degradation of p65, thereby inhibiting the NF-κB signaling pathway that is essential for the self-renewal of TICs. Conversely, ACTBL2 competes with RBCK1 for binding to MTSS1, stabilizing p65 and thereby rescuing the tumorigenic activity that MTSS1 suppresses [62]. In the context of lung adenocarcinoma, MTSS1 interacts with the E3 ligase AIP4 to monoubiquitinate PD-L1 at lysine 263, which targets it for lysosomal degradation. Additionally, MTSS1 promotes the lysosomal degradation of PD-L1 through a mechanism that is independent of CMTM6 [60].

## Conclusions and future perspectives

MTSS1’s most significant identified function to date is its effect on cancer cells, increasing the migratory and metastatic capacity when downregulated. In addition, MTSS1 interacts with and modulates a wide range of cellular processes affecting the actin cytoskeleton, cell shape and mobility, cell differentiation, signaling processes, and protein turnover (Fig. 1). Consequently, the pathological effects associated with MTSS1 dysfunction are diverse, spanning from cancer progression to metastasis control and inflammation modulation. Pathologic outcomes relate to the expression levels of MTSS1, particularly high or low expression levels in relation to the physiologic levels in the same cell type, rather than de novo mutations affecting function. There are no reports on mutations in MTSS1 leading to pathologic outcomes. Hence, intact MTSS1 must be essential during embryogenesis. Even though mice lacking MTSS1 survive the embryonic stage, they still suffer from severe kidney and neurological defects. One explanation for why MTSS1 ablation is not embryonically lethal might be linked to the compensatory expression of the other I-BAR proteins, although this possibility has not yet been studied.

The literature on MTSS1’s role as a metastasis suppressor is rich, predominantly showing that MTSS1 blocks metastasis-associated migration. Although current knowledge points to the role of MTSS1 in cytoskeletal modulation, lipid membrane shaping, and lamellipodia formation, the mechanism underlying metastasis suppression deserves more attention. There is still much to learn from studies on the role of MTSS1 in diverse physiologic contexts. Although preventing late-stage spread in advanced cancer is the most important future aim of drug development, MTSS1’s functions require clarification before considering anti-cancer therapies based on this protein. It is also important to ensure that targeting MTSS1 will not have any adverse effects on the brain, taking into account the important roles of MTSS1 in neuronal connectivity. We do not yet understand the details of how MTSS1 is antimetastatic. Intensified studies on the functions and pathologic effects of MTSS1 are likely to reveal important mechanisms in protein–lipid membrane interactions and possible strategies to prevent metastasis.

## Data Availability

Not applicable.
